# The Anterior Piriform Cortex and Predator Odor Responses: Modulation by Inhibitory Circuits

**DOI:** 10.3389/fnbeh.2022.896525

**Published:** 2022-04-28

**Authors:** Mutsumi Matsukawa, Masaaki Yoshikawa, Narumi Katsuyama, Shin Aizawa, Takaaki Sato

**Affiliations:** ^1^Division of Anatomical Science, Department of Functional Morphology, Nihon University School of Medicine, Itabashi, Japan; ^2^Cognitive Neuroscience Section, Primate Research Institute, Kyoto University, Inuyama, Japan; ^3^Biomedical Research Institute, National Institute of Advanced Industrial Science and Technology, Ikeda, Japan

**Keywords:** predator odor, stress, innate response, relaxant, feedforward inhibition, anterior piriform cortex, monoaminergic neuromodulation system

## Abstract

Rodents acquire more information from the sense of smell than humans because they have a nearly fourfold greater variety of olfactory receptors. They use olfactory information not only for obtaining food, but also for detecting environmental dangers. Predator-derived odor compounds provoke instinctive fear and stress reactions in animals. Inbred lines of experimental animals react in an innate stereotypical manner to predators even without prior exposure. Predator odors have also been used in models of various neuropsychiatric disorders, including post-traumatic stress disorder following a life-threatening event. Although several brain regions have been reported to be involved in predator odor-induced stress responses, in this mini review, we focus on the functional role of inhibitory neural circuits, especially in the anterior piriform cortex (APC). We also discuss the changes in these neural circuits following innate reactions to odor exposure. Furthermore, based on the three types of modulation of the stress response observed by our group using the synthetic fox odorant 2,5-dihydro-2,4,5-trimethylthiazoline, we describe how the APC interacts with other brain regions to regulate the stress response. Finally, we discuss the potential therapeutic application of odors in the treatment of stress-related disorders. A clearer understanding of the odor–stress response is needed to allow targeted modulation of the monoaminergic system and of the intracerebral inhibitory networks. It would be improved the quality of life of those who have stress-related conditions.

## Introduction

Rodents use odors as cues for evaluating their surroundings, for locating food, social interactions and breeding, and the detection of threats in the environment. With respect to threat detection, the primary focus of this review, rapid and accurate detection of threat in its proximity is critical for survival (e.g., cat, fox, and ferret). The odors produced by these predators, known as kairomones, which are the interspecific chemical signals that cause a disadvantage to the source of release, rapidly induce fear and stress responses in rodents (Vernet-Maury et al., 1984; Apfelbach et al., 2005; Takahashi, 2014; Janitzky et al., 2015; Masuo et al., 2021). While the stress responses are critical for survival, dysregulation of these responses can result in psychiatric disorders in humans, such as anxiety and mood disorders, phobias, and post-traumatic stress disorder (PTSD) (Rosen et al., 2008; Staples, 2010; Zoladz et al., 2012; Wilson et al., 2014; Dopfel et al., 2019).

There is an extensive neural network that responds to predator odors. A constituent odorant of fox feces, 2,5-dihydro-2,4,5-trimethylthiazoline (TMT), which mimics the anal gland secretions of the fox, is one of the most effective at inducing innate stress responses in rodents (Vernet-Maury et al., 1984; Day et al., 2004). The detection of TMT appears to be predominantly through the piriform cortex (PC) *via* the olfactory bulb (OB) which receives input signals from olfactory sensory neurons activated in the olfactory epithelium (OE) (Takahashi, 2014). Although the anterior piriform cortex (APC) has known to involve both detection of TMT and induction of TMT-induced stress responses, the underlying mechanisms including the related neural network and modulatory system have thought to be different (Kobayakawa et al., 2007). One of the immediate early genes, c-fos, is a biomarker of neural activity, and is associated with stress responses. Based on c-fos expression, it has been shown that various brain regions, including the APC, medial part of the bed nucleus of the stria terminalis (mBST), and amygdalopiriform transition area (AmyPir), are involved in the stress response to predator odor (Day et al., 2004; Kobayakawa et al., 2007; Janitzky et al., 2009; Kondoh et al., 2016). In this review, we describe the effects of odor in the modulation of stress responses, focusing on the role of the inhibitory circuits in the APC. We also discuss how a better understanding of the regulation of stress responses is important for treating stress-related illnesses.

## Stress and Odor

### Responses to Stress and Fear

There are autonomic, endocrine, and behavioral responses to fear and stress. The autonomic nervous system, which provides a rapid and short-term response to stress, raises heart rate and blood pressure, enabling a rapid response to threats (Ulrich-Lai and Herman, 2009). The endocrine response, which involves activation of the hypothalamic–pituitary–adrenal (HPA) axis, is less rapid, but is sustained for a longer period (Ulrich-Lai and Herman, 2009). Activation of the HPA axis stimulates endocrine release, including adrenocorticotropic hormone (ACTH), cortisol, and corticosterone, which can be used as biomarkers of stress. In addition, behavioral responses to stress and fear may include freezing, attack/avoidance and other behaviors, and these too can be used to assess anxiety.

Moreover, monoaminergic neuromodulation system has thought to be involved in the regulation of stress-related responses. Although definitive biomarkers of PTSD remain elusive, dysregulation of various neurotransmitter systems has been implicated in the disorder (Arora et al., 1993; Geracioti, Baker et al., 2001; Krystal and Neumeister, 2009; Southwick and Charney, 2012). Monoamines, including dopamine (DA), norepinephrine (NE), and serotonin (5-HT), are synthesized in specific regions of the brain and regulate neural activities in numerous target regions. Moreover, predator odors can increase the release of monoamines in the mouse brain (Hayley et al., 2001; Smith et al., 2006) and activate the locus coeruleus (LC), from which noradrenergic fibers innervate the cerebral cortex, amygdala, and hippocampus (Day et al., 2004; Curtis et al., 2012; Isosaka et al., 2015; Janitzky et al., 2015). These monoamines play important roles in behavioral responses to innate and learned fears and stresses by modulating motor control, motivation, reward, learning, and associative memory.

### Modulation of Stress by Odor

#### Innate Responses

We previously showed that natural odors can be classified into three types: (1) odors that cause innate fear/stress reactions (innate stressors) such as predator odors; (2) odors that innately reduce stress signaling (innate relaxants) such as rose and hinokitiol odors; and (3) odors that do not affect stress responses (neutral odors) such as caraway odor (Matsukawa et al., 2011; Murakami et al., 2012). Next, we describe the effects and associated systems for each type of odor.

#### Innate Stressors

In a number of studies, predator odors, such as those of cats and ferrets, have also been used to activate stress pathways in rats and mice (Takahashi, 2014). Despite the absence of a real threat, TMT activated stress responses, which are to increase plasma ACTH levels and to heighten the activities in the predator odor-related neural pathways (Day et al., 2004; Matsukawa et al., 2011). In addition to TMT, predator urines have been shown to induce neural activation in the main olfactory system (MOS) *via* the main OB (MOB) (Takahashi, 2014). Furthermore, stress responses are strongly linked with learning and memory processes, and thus may adapt to predator odors over time (Staples, 2010). The stimulus intensity of predator odors (concentration and repeated administration) is known to impact habituation and extinction (Takahashi et al., 2005). Moreover, neonatal exposure to TMT odor in mice can reduce avoidance, immobility and freezing behaviors in adulthood (Hacquemand et al., 2010). In addition to odors from external threats, a number of studies have shown that stressed animals can release odors called warning pheromones that cause anxiety-related behaviors in other conspecifics in their proximity (Masuo et al., 2021).

#### Innate Relaxants and Neutral Odors

Conversely, several odors have been shown to improve mood or signs of stress or anxiety (Haze et al., 2002; Lehrner et al., 2005; Ito et al., 2009; Masuo et al., 2021). In addition, some odors can decrease responses to stressor odors. For example, rose odor reduces plasma ACTH levels during TMT-induced stress responses by decreasing c-fos upregulation-associated neural activation in the ventrorostral part of the APC (APCvr) and the mBST in mice (Table 1; Matsukawa et al., 2011). Notably, robust odor-induced feedforward inhibitory signals are sent from the APCvr to the entire APC (Ishikawa et al., 2007). Hinokitiol odor, a woody scent, but not S(+)-carvone (caraway odor), prevents the TMT-induced upregulation of plasma ACTH by increasing c-fos-associated neural activation in the lateral part of the BST (lBST) when presented in combination with TMT odor (Table 1; Murakami et al., 2012). This demonstrates that animals have innate responses to specific odors, and that some odors, such as rose and hinokitiol odors, reduce stress responses without prior learning, while others, like caraway odor, are innately neutral and do not significantly impact the stress response.

**TABLE 1:** Changes in the number of c-fos-positive cells in each brain region following each odor presentation (upper part) and following each electrical stimulation (lower part).

	Plasma	c-fos positive cells						
		
	ACTH	OB	APCvr	APCd	mBST	lBST	AmiPir	PPC
TMT	++	++	++	++	++	n.s.	++	n.s.
TMT + rose	n.s.**	++	++*	++	n.s.**	n.s.	−	−
TMT + hinokitiol	n.s.**	−	++	++**	++	++**	−	−
TMT + caraway	++	−	++	++	++	n.s.	−	−
TMT + habitat odor	n.s.***	−	++*	++	+**	n.s.	−	−

Significant differences compared with TMT; *P < 0.05, **P < 0.01, ***P < 0.001;								
n.s., no significant difference (*P* ≥ 0.05); –, no data available.								

mOB electrical stimulation	−	−	+++	+++	n.s.	n.s.	n.s.	+++
dlOB electrical stim	−	−	+	+++	n.s.	n.s.	n.s.	+++
mOB and dlOB electrical stimulation	−	−	+++**	+++	+*	+	+++***	+++

Significant differences compared with each OB stimulation; *P < 0.05, **P < 0.01, ***P < 0.001;								
n.s., no significant difference (*P* ≥ 0.05); –, no data available.								

Apparently, there are multiple mechanisms that induce the innate inhibitory response. When combined with TMT odor, hinokitiol odor may activate the olfactory system broadly and robustly, thereby making the specific effect of TMT impossible to distinguish at the neural level (Table 1; Murakami et al., 2012). These findings suggest that there may be at least two distinct mechanisms that reduce TMT odor-induced stress responses: (1) direct and selective mechanisms that inhibit TMT odor-induced activation of stress-related networks, as seen with rose odor; and (2) mechanisms that obscure the selective effect of TMT odor, as seen with hinokitiol odor.

#### Conditioned Responses

Experiences can change the innate reactions. We previously demonstrated that even an artificial odor, classified as a neutral odor, could alleviate the predator odor-induced stress responses in adulthood in mice when they experience and are habituated to the artificial odor early in life (Matsukawa et al., 2016). This suggests that animals can be conditioned to experience stress-relieving effects from odors later in life when experienced in the absence of a real threat. This also means that experience can modify innate reactions. When combined with a habitat odor, TMT odor-induced neural activation is reduced in the APCvr and the mBST, similar to the effect of rose odor (Table 1; Matsukawa et al., 2016). These stress-reducing effects suggest that a selective inhibitory system is present in brain regions that participate in predator odor-induced stress responses.

## The Mechanisms of Odor-Induced Stress Reactions: Olfaction and Stress Responses

### Neural Substrate of Olfactory Information Processing

**FIGURE 1:**
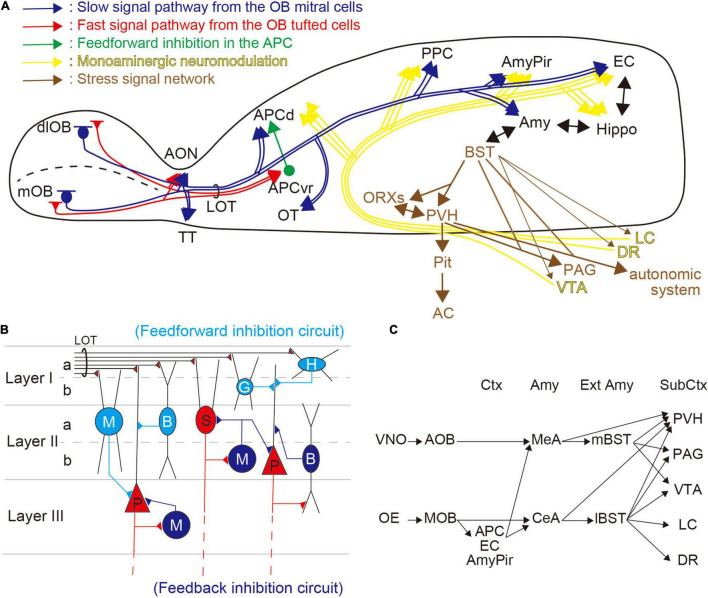
Olfactory information processing and stress-related networks. **(A)** Overview of the regulation of the odor–stress response. Distinct olfactory bulb (OB) projection neurons are shown in red [tufted cells (TCs)] and blue [mitral cells (MCs)] lines, and show fast and slow pathways, respectively. TCs project to the anterior olfactory nucleus (AON) and the ventrorostral part of the anterior piriform cortex (APCvr). MCs innervate the AON, the olfactory tubercle (OT), the APCd, the posterior piriform cortex (PPC), the amygdalopiriform transition area (AmyPir), the entorhinal cortex (EC), and the amygdala (Amy). Feedforward inhibitory circuit from the APCvr to the APCd is shown as a green line. Stress-related networks are illustrated in brown; from the bed nucleus of the stria terminalis (BST), the information passes to the hypothalamus (PVH and ORXs) where it can activate the hypothalamic–pituitary–adrenal (HPA) axis. Many brainstem regions, including the periaqueductal gray (PAG) and autonomic system, receive information from the BST and hypothalamus. The yellow lines show monoaminergic modulatory networks. DA projections from the ventral tegmental area (VTA), NE projections from the locus coeruleus (LC), and serotonin (5-HT) projections from the dorsal raphe (DR) are distributed widely in the brain. **(B)** Inhibitory pathways in the piriform cortex (PC). In the PC, olfactory information from the OB is obtained *via* the lateral olfactory tract (LOT). Horizontal cells (H), neurogliaform cells (G), bitufted cells (B), and multipolar cells (M), which are inhibitory interneurons in the superficial layer (illustrated in light blue), have feedforward connections to projection neurons [semilunar cells (S) and pyramidal cells (P); illustrated in red] in the PC. Inhibitory cells in mid to deep layers (B and M; illustrated in blue) feedback onto S and P cells. **(C)** Putative networks for odor-induced stress responses. The accessory olfactory system (AOS) from the vomeronasal organ (VNO) provides inputs to the extended amygdala (Ext Amy) *via* the medial amygdala (MeA). In contrast, the main olfactory system (MOS) from the olfactory epithelium (OE) provides inputs to the Ext Amy directly from the central amygdala (CeA) and indirectly *via* olfactory cortices, including the APC, EC, and AmyPir. Both the Amy and Ext Amy have projections to subcortical regions (Sub Ctx) including the PVH, PAG, VTA, LC, and DR.

In mammals, the olfactory system is composed of two distinct pathways—the MOS and the accessory olfactory system (AOS). The MOS is considered to be primarily involved in the detection of environmental cues such as foods and predators (Takahashi, 2014; Masuo et al., 2021), whereas the AOS is more involved in the detection of intraspecific chemical stimuli such as pheromones (Wyatt, 2017; Mohrhardt et al., 2018). The MOS comprises the main OE, which expresses odorant and trace amine-associated receptors. These detect environmental cues and convey the information to the olfactory cortex [APC, posterior piriform cortex (PPC), AmyPir, entorhinal cortex (EC)] and other areas, including the amygdala, to modify physiological and behavioral responses (Figure 1A; Takahashi, 2014; Isosaka et al., 2015; Kondoh et al., 2016). Both the amygdala and the EC have bidirectional innervation to the hippocampus, and bidirectional connections exist between the amygdala and extended amygdala, including the BST.

### Relationship Between Olfaction and Stress Responses

#### The Piriform Cortex

The PC, which is the largest area in the primary olfactory cortex, consists of three layers (Layers I, II, and III) and is divided into two regions, the APC and the PPC. These two regions have different roles in olfactory information processing. The APC encodes odorant information, perception, and odor-associated values, while the PPC encodes associated information (e.g., odor similarity, quality) (Litaudon et al., 2003; Kadohisa and Wilson, 2006; Calu et al., 2007; Roesch et al., 2007). A recent study showed that the PPC has a key role in spatial representation, including the formation of odor–place associations and guided olfactory-based spatial navigation (Poo et al., 2022). The APC and the PPC are also neuroanatomically different—the APC receives greater input from olfactory regions than the PPC, while the latter receives comparatively more projections from the hippocampus and cerebral nuclei (Wang et al., 2020). In addition, the APC can be subdivided into two distinct parts, the APCvr and the dorsal part of the APC (APCd), by morphological analysis (Ekstrand et al., 2001). In the MOB, there are two distinct projection neurons—mitral cells (MCs) and tufted cells (TCs). The axonal projections of MCs and TCs differ, with MCs sparsely projecting to the APCd, and the TCs robustly projecting to the APCvr (Figure 1A; Igarashi et al., 2012).

Despite its relatively simple trilaminar cortical structure, the PC has many inhibitory interneurons for both feedforward and feedback connections (Figure 1B; Gavrilovici et al., 2010; Suzuki and Bekkers, 2012; Bekkers and Suzuki, 2013; Large et al., 2016). The feedforward inhibition usually occurs when excitatory inputs activate inhibitory cells, which then inhibit postsynaptic excitatory neurons (Kee et al., 2015). In contrast, feedback inhibition generally occurs when excitatory neurons activate inhibitory cells that then recurrently inhibit them (Kee et al., 2015). In the PC, the interneurons responsible for feedforward inhibition are localized superficially (Layers I–II), and those responsible for feedback connections are distributed in deep layers (Layers II–III) (Figure 1B).

#### The Bed Nucleus of the Stria Terminalis

The BST, known as the extended amygdala, has an important role in stress and fear responses. This forebrain region, made up of more than 10 subregions, comprises distinct neuroanatomical and neurochemical populations. AOS information from the vomeronasal organ (VNO) provides input to the mBST *via* the medial amygdala (MeA), and MOS information from the OE provides input to both the mBST, *via* the MeA, and the lBST, *via* the central amygdala (CeA). In addition, the BST outputs project to the hypothalamus [e.g., the paraventricular hypothalamic nucleus (PVH), orexin neurons (ORXs)] and brainstem regions [e.g., the periaqueductal gray (PAG), and the origins of monoaminergic innervation, including the ventral tegmental area (VTA), dorsal raphe nucleus (DR), and LC] (Figure 1C; Takahashi, 2014; Fox et al., 2015; Lebow and Chen, 2016). The HPA axis may be stimulated, *via* the mBST, following presentation of the predator odor (Kobayakawa et al., 2007). In addition, the BST appears to play a role in conditioned fear responses with a temporal aspect (Goode and Maren, 2017). Moreover, recent studies suggest that the BST modulates the response to weak, uncertain threats (Glover et al., 2020; Bruzsik et al., 2021).

#### Inhibitory Circuits and the Piriform Cortex

2,5-Dihydro-2,4,5-trimethylthiazoline odor information is conveyed from the MOB to the APC by several neural networks. The OE innervates spatially distinct glomeruli forming mirror-image maps in the MOB (Nagao et al., 2000; Uchida et al., 2000; Inaki et al., 2002; Mori et al., 2006; Matsumoto et al., 2010; Murthy, 2011). We demonstrated that stress-related neural activities were induced only following simultaneous stimulation in the mirror-image-organized medial and dorso-lateral walls of the MOB (mOB and dlOB, respectively), but not following stimulation of the mOB or dlOB individually (Table 1; Matsukawa et al., 2020). The TCs, whose firing rates during burst discharges are about 100 Hz, provide rapid input to the APCvr (fast pathway), whereas the MCs, whose firing rates during burst discharges are about 40 Hz, provide slow input to the APCd (slow pathway) (Figure 1A; Nagayama et al., 2004; Igarashi et al., 2012). We previously suggested that the association of beta-band (15–40 Hz) oscillations from MCs in both mOB and dlOB should be needed for stress-induced activation of the APCd (Matsukawa et al., 2020). In addition, through the inhibitory circuit from the APCvr, the fast pathway component can regulate the association of oscillatory activities in the APCd (Ishikawa et al., 2007; Sato et al., 2008). It has been shown that the integration and processing of the mOB and dlOB inputs in the APC are important for the expression of what is termed the odor–stress response (Matsukawa et al., 2020). Furthermore, we showed that the selective inhibitory system involved in reducing the predator odor-induced stress response is likely to be in the APC rather than in the OB (Matsukawa et al., 2011, 2016, 2020).

## The Therapeutic Importance of Odor Modulation of Stress Responses

Monoamine neurotransmitters have been the primary target of therapeutic strategies for the treatment of neuropsychiatric diseases such as PTSD (Krystal and Neumeister, 2009). In animal models of PTSD, lower 5-HT and elevated NE levels have been observed in the prefrontal cortex and hippocampus (Wilson et al., 2014). In addition, we demonstrated changes in hippocampal NE concentrations following TMT presentation (Matsukawa et al., 2016). These studies suggest that increased hippocampal NE concentrations are important in the stress response following exposure to a life-threatening event/cue. Higher concentrations of NE in the hippocampus have also been shown to affect the regulation of stress responses and learning under stress (de Kloet et al., 2005; Joëls et al., 2006; Ulrich-Lai and Herman, 2009). Moreover, hippocampal NE concentrations, under stress conditions, have also been shown to affect memory consolidation *via* the amygdala (Roozendaal et al., 2009; Galliot et al., 2010; Hubert and Muly, 2014). Recent studies have shown that medial prefrontal areas, including the prelimbic, infralimbic, and anterior cingulate cortices, can regulate the response to predatory threats (Glover et al., 2020; de Lima et al., 2022). Dopaminergic innervation from the VTA has also been reported in these brain regions, where the number of synapses is regulated by DA in Layer I *via* D1 receptor as well as by NE in Layer II/III (Imai et al., 2004). Together, these observations suggest that monoamines, which can modify the neural activities in broad regions of the brain, participate in odor-induced stress responses.

In addition to monoaminergic modulation, there are many intrinsic inhibitory circuits that use a variety of inhibitory neurotransmitters in the APC (Gavrilovici et al., 2010; Suzuki and Bekkers, 2012; Bekkers and Suzuki, 2013; Large et al., 2016), as well as inhibitory connections among subregions in the APC (Ishikawa et al., 2007; Sato et al., 2008). However, many issues remain to be addressed, such as whether the selective inhibition system in the APC is a specific response to TMT odor or common to other predator odors. A better understanding of the inhibitory mechanisms that selectively suppress the odor-induced stress response in the APC may be important for the therapeutic modulation of odor–stress relationships.

## Conclusion and Future Studies

Odor-induced stress responses, which involve autonomic, endocrine, and behavioral responses, can be modulated by innate and conditioned odors. This modulation of the response to stress-inducing stimuli such as predator odors likely involves inhibitory pathways in the APC. These pathways have potential as therapeutic targets for conditions such as chronic stress, anxiety, phobias, and PTSD. However, many questions remain unresolved, including which inhibitory neural circuits and inhibitory neurotransmitters are involved in a condition-dependent manner. Moreover, a recent study suggests that transient receptor potential ankyrin 1 (TRPA1) participates in predator odor-evoked innate fear responses (Wang et al., 2018). TRPA1, a chemoreceptor for noxious stimuli, such as formaldehyde (McNamara et al., 2007), has an important role in nociception (Story et al., 2003). In addition, a recent study suggests that innate fear stimuli orchestrate hypothermia and anti-hypoxia *via* TRPA1 activation (Matsuo et al., 2021). Elucidation of stress-related neural circuits, their selective inhibitory systems, and related chemoreceptors is necessary for the future development of effective therapeutic interventions.

## Author Contributions

MM planned and conducted most of these studies with co-authors. MY planned and conducted collection of brain samples from experimental animals and investigation for c-fos study. NK planned and conducted experiments for effects of electrical stimulations in mirror-arranged glomeruli in the OB. SA designed and performed the collection of plasma samples and investigation of odor-induced stress responses. TS designed and conducted studies on the effects of different odors on TMT-induced stress-related responses in mice. MM and TS wrote the manuscript. All authors analyzed the data and discussed the results.

## Conflict of Interest

The authors declare that the research was conducted in the absence of any commercial or financial relationships that could be construed as a potential conflict of interest.

## Publisher’s Note

All claims expressed in this article are solely those of the authors and do not necessarily represent those of their affiliated organizations, or those of the publisher, the editors and the reviewers. Any product that may be evaluated in this article, or claim that may be made by its manufacturer, is not guaranteed or endorsed by the publisher.
